# Efficacy and safety of sodium-glucose cotransporter 2 (SGLT2) inhibitors in patients with acute heart failure: a systematic review and meta-analysis

**DOI:** 10.3389/fcvm.2024.1388337

**Published:** 2024-09-11

**Authors:** Jingjin Hou, Li Ren, Qingbin Hou, Xiaodong Jia, Zhu Mei, Jiaxin Xu, Zheming Yang, Yiming Li, Chenghui Yan

**Affiliations:** State Key Laboratory of Frigid Zone Cardiovascular Disease, Cardiovascular Research Institute and Department of Cardiology, General Hospital of Northern Theater Command, Shenyang, China

**Keywords:** acute heart failure, SGLT2 inhibitors, all-cause mortality, quality of life, renal function, adverse events

## Abstract

**Background:**

The effectiveness and safety of a novel class of hypoglycemic medications known as sodium-glucose cotransporter 2 (SGLT2) inhibitors have not been completely established in relation to acute heart failure (AHF). Consequently, we sought to compare the prognostic and safety outcomes of patients administered SGLT2 inhibitors for the treatment of AHF.

**Methods:**

An extensive search of the Web of Science, PubMed, and EMBASE was conducted for randomized controlled trials and observational studies that have evaluated the use of SGLT2 inhibitors in AHF from the inception of these drugs to the present. We compiled data related to cardiovascular safety and prognosis. Aggregated risk ratios (RR), mean differences (MD), or standardized mean differences (SMD) were generated for all outcomes, with 95% confidence intervals (CIs), to evaluate the predictive significance of SGLT2 inhibitors in patients with AHF.

**Results:**

We identified 4,053 patients from 13 studies. Patients experienced a substantial reduction in all-cause mortality (RR = 0.82, 95% CI: 0.70–0.96, *P* = 0.01), readmission rates (RR = 0.85, 95% CI: 0.74–0.98, *P *= 0.02), the number of heart failure exacerbation events (RR = 0.69, 95% CI: 0.50–0.95, *P* = 0.02), and the number of rehospitalization events due to heart failure (RR = 0.71, 95% CI: 0.58–0.86, *P* < 0.05) in the SGLT2 inhibitors-treatment group compared to a placebo or standard care (control group). SGLT2 inhibitors improved patient quality of life (SMD = −0.24, 95% CI: −0.40 to −0.09, *P* = 0.002). SGLT2 inhibitors were associated with enhanced diuresis in patients with AHF (MD = 2.83, 95% CI: 1.36–4.29, *P* < 0.05). Overall, treatment with SGLT2 inhibitors significantly reduced the level of serum NT-proBNP (MD = −497.62, 95% CI: −762.02 to −233.21, *P* < 0.05) and did not increase the incidence of adverse events (RR = 0.91, 95% CI: 0.82–1.01, *P* = 0.06).

**Conclusions:**

This meta-analysis suggests that treatment with SGLT2 inhibitors is associated with a better prognosis in patients with AHF than in patients not treated with SGLT2 inhibitors. It is safe and effective to initiate SGLT2 inhibitors in patients with AHF.

**Systematic Review Registration:**

https://www.doi.org/10.37766/inplasy2024.9.0015, identifier (INPLASY202490015).

## 1 Introduction

Heart failure (HF) is a clinical syndrome characterized by signs and symptoms resulting from abnormalities in heart structure and function. Elevated natriuretic peptide levels or apparent pulmonary or systemic congestion frequently accompany these abnormalities (1). Patients with HF face substantial medical expenses, a decreased standard of living, and increased rates of morbidity and mortality. Current estimates indicate that the global HF patient population exceeds 640,000 individuals (2), imposing a significant burden on healthcare expenditure worldwide. Acute heart failure (AHF) is a condition in which the heart suddenly becomes unable to pump blood efficiently and can be caused by a variety of factors, including myocardial infarction, arrhythmia, or infection. AHF is a prevailing cause of unplanned hospital admissions in patients aged >65 years worldwide. Acute decompensated heart failure (ADHF) is a form of AHF that refers to patients with chronic heart conditions characterized by a sudden worsening of their condition that necessitates intensive treatment. AHF is a broader term that can include any cause of sudden decline in heart function; therefore, ADHF is a subtype of AHF (3). Primary treatment objectives are a reduction in congestion and optimization of guideline-directed medical therapies. While diuretics may ameliorate congestion during hospitalization for AHF, they do not enhance the prognosis. Consequently, AHF is associated with adverse outcomes.

Sodium-glucose cotransporter 2 (SGLT2) inhibitors are a new type of antidiabetic medication that includes canagliflozin, dapagliflozin, empagliflozin, ertugliflozin, and henagliflozin. According to current national and international guidelines, individuals with type 2 diabetes who also have HF, chronic renal disease, cardiovascular illness, or cardiovascular risk factors should take SGLT2 inhibitors (4). This recommendation stems from the cardiac and renal benefits that these inhibitors have shown in large-scale clinical studies, including a reduction in hospitalization risk for patients with stable chronic HF and reduced left ventricular ejection fraction (5). Dapagliflozin and empagliflozin, as representatives of SGLT2 inhibitors, have emerged as the first class of therapeutic agents to improve the prognosis of HF across a range of ejection fractions.

Randomized clinically controlled trials (RCTs) and meta-analyses of the results of trials in patients with HF have shown that SGLT2 inhibitors improve cardiovascular outcomes in patients with chronic HF, irrespective of their diabetes status (6). However, whether SGLT2 inhibitors have a clinical benefit in patients with AHF is being explored. Several multicenter RCTs have examined the efficacy of SGLT2 inhibitors in patients with AHF. The EMPA-RESPONSE-AHF study (7) highlighted the safety and potential benefits of the early use of SGLT2 inhibitors in patients with AHF, which reduced the combined endpoint of HF exacerbation, HF rehospitalization, or 60-day death but did not affect diuretic response in patients with AHF. SGLT2 inhibitors significantly lower the renal glucose threshold, reduce reabsorption, and promote the excretion of large amounts of glucose in urine. In addition to concerns regarding adverse events, including urinary infection and diabetic ketoacidosis, research is increasingly focused on the urinary efficiency of SGLT2 inhibitors. Until now, SGLT2 inhibitors have been associated with enhanced diuresis in patients with AHF, for example, in the DICTATE-AHF trial (8). To determine whether SGLT2 inhibitors improve the prognosis of patients with AHF, we performed an extensive literature search, incorporating all pertinent observational studies and randomized controlled trials. This study aimed to clarify the safety and clinical effectiveness of SGLT2 inhibitors in treating AHF.

## 2 Materials and methods

### 2.1 Study design and search strategy

Up to April 10, 2024, two separate researchers (J.H. and J.X.) conducted a comprehensive search of PubMed, Embase, and Web of Science databases to identify studies that examined the efficacy of SGLT2 inhibitors in patients with AHF, without restrictions on language or sample size. In the identified trials, patients admitted with AHF were stratified into two groups: those receiving a single SGLT2 inhibitor and those receiving either placebo or conventional treatment. The following search strategy was employed: (“SGLT2 inhibitor” OR “dapagliflozin” OR “empagliflozin” OR “canagliflozin” OR “ertugliflozin” OR “henagliflozin”) AND (“acute heart failure” OR “acute decompensated heart failure”). The reference lists of selected articles were manually examined to identify additional relevant studies. Duplicate publications, studies with incomplete data, and studies in which the participants or interventions did not align with the defined inclusion criteria were excluded.

### 2.2 Inclusion and exclusion criteria

The inclusion criteria for the studies were: (1) RCTs or observational study design; (2) a focus on AHF; (3) inclusion of an experimental group receiving a single SGLT2 inhibitor regardless of the initiation time of the intervention; (4) patients with or without diabetes; and (5) those that reported predetermined efficacy and safety outcomes. The exclusion criteria were (1) studies in which patients did not have AHF, and (2) studies in which the experimental group received multiple SGLT2 inhibitors.

### 2.3 Data extraction

From each selected study, the following data were separately collected by two investigators (Z.M. and L.R.). Conflicts in data extraction were resolved by a third investigator (Y.L.). The following data were extracted: first author, publication year, study design, number of participants in the intervention and control groups, specific SGLT2 inhibitor used, participant age (mean or median), duration of follow-up, primary outcomes, and secondary outcomes. The efficacy outcomes included all-cause mortality, frequency of readmission events, heart failure exacerbation (HFE) events, rehospitalization due to HF, deaths attributable to cardiovascular causes, quality of life assessments, and renal function evaluations. Safety outcomes included the total number of adverse events, including urinary tract infections, ketoacidosis, hypotension, hypoglycemia, and acute kidney injury.

### 2.4 Study quality assessment

Two researchers (Z.Y. and L.R.) separately evaluated the quality of the included studies. Disagreements were resolved through discussion, and a third reviewer was consulted if necessary. We used the Cochrane Risk of Bias instrument (9) for RCTs and the Newcastle-Ottawa Scale (NOS) (9) for observational studies. Each type of bias was classified as presenting a “low”, “unknown,” or “high” risk of bias. For observational studies, we employed the NOS, which focuses on three aspects: selection, comparability, and outcomes. When the risk of bias was “low”, it was awarded one point (“*”), while “unclear” or “high” risk of bias received no points (“-”). Studies achieving a NOS score higher than 6 were considered to be of high quality.

### 2.5 Statistical methods

Review Manager Version 5.3 (RevMan) and Stata 18.0 were used for statistical analyses. Risk ratio (RR), mean difference (MD), or standardized mean difference (SMD) were used to represent outcome measures in the treatment vs. control groups. The initial analysis entailed a heterogeneity test using the chi-square test to calculate I^2^ and detect any heterogeneity between the included studies. The appropriate effect model was then selected based on the I^2^ or *P*-value. When considerable heterogeneity was found, that is, an I^2^ value exceeding 50% or a chi-square *P*-value lower than 0.10, the use of a random-effects model was justified. Conversely, a fixed-effects model was used when warranted. Furthermore, the origins of heterogeneity were analyzed. In RCTs, a segment of the population in the SOLOIST-WHF study (10) was allocated the SGLT2 inhibitor therapy only after discharge. In an observational study by Nakagaito et al. (11), the decision to continue treatment with the SGLT2 inhibitor was made only at discharge. Consequently, when conducting a combined effect size analysis incorporating the results of these two studies, a sensitivity analysis excluding these two studies was performed to examine how these exclusions affected the outcome measures. Additionally, we utilized Stata software for sensitivity analysis and Egger's test for certain outcomes.

## 3 Results

### 3.1 Literature search

**Figure 1:**
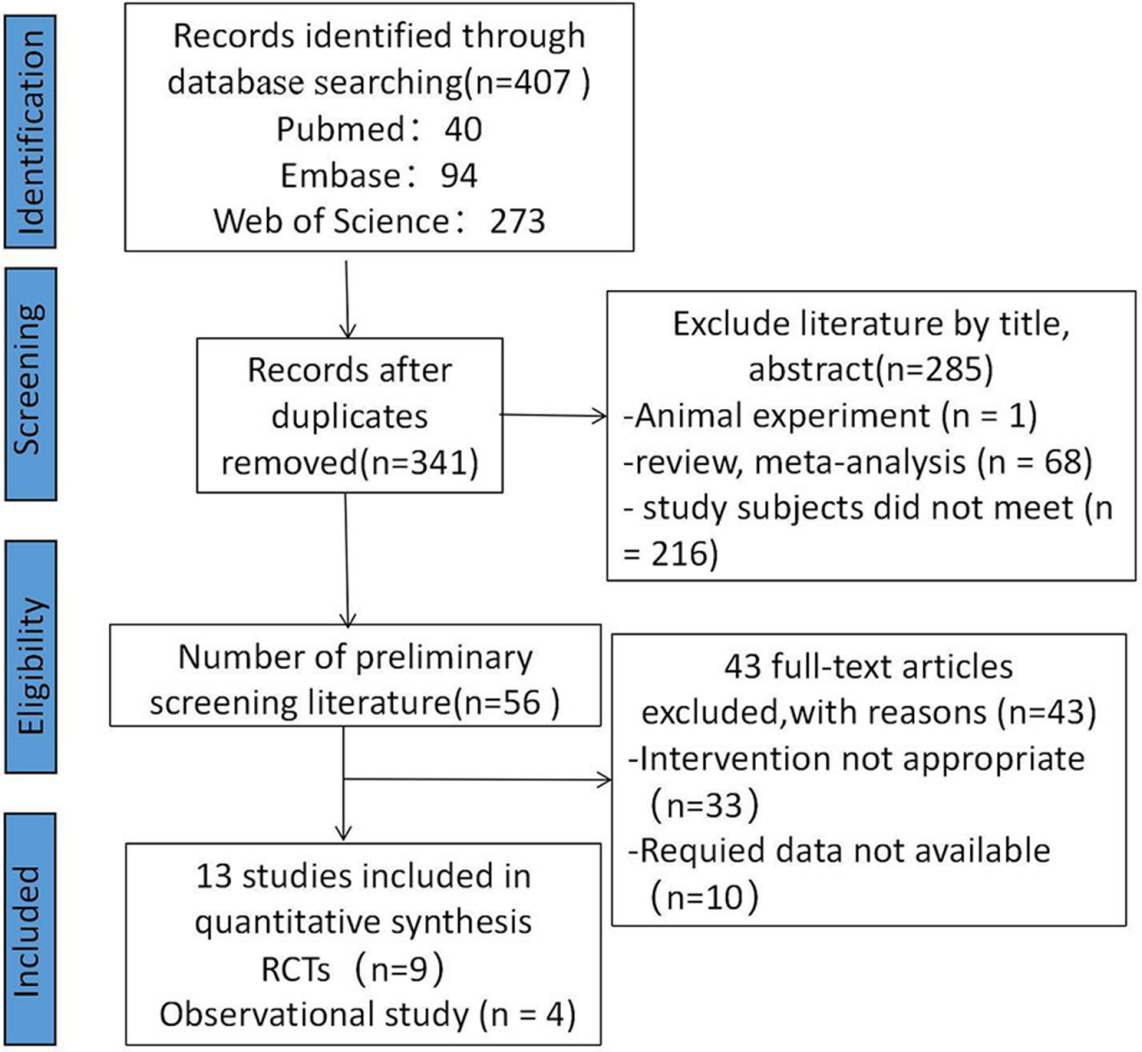
PRISMA flow diagram of study selection.

The PRISMA diagram representing the study selection process is depicted in Figure 1. The search produced a total of 407 individual articles. After screening titles and abstracts for relevance to the inclusion criteria and removing 66 duplicate reports, 285 articles were excluded. Following a full-text review, 13 studies that met the inclusion criteria were identified (7, 8, 10–20). These included nine RCTs (7, 8, 10, 12–17) and four observational studies (11, 18–20). The characteristics of the included studies are summarized in Supplementary Table 1. The DICTATE-AHF trial (8) initially included 238 study participants; however, because of a primary investigator withdrawing and a participant withdrawing on consent day 1, among other reasons, there were 118 patients in the SGLT2 inhibitor intervention group and 116 patients in the control group at the time of the final study.

### 3.2 Risk of bias assessment

**Figure 2:**
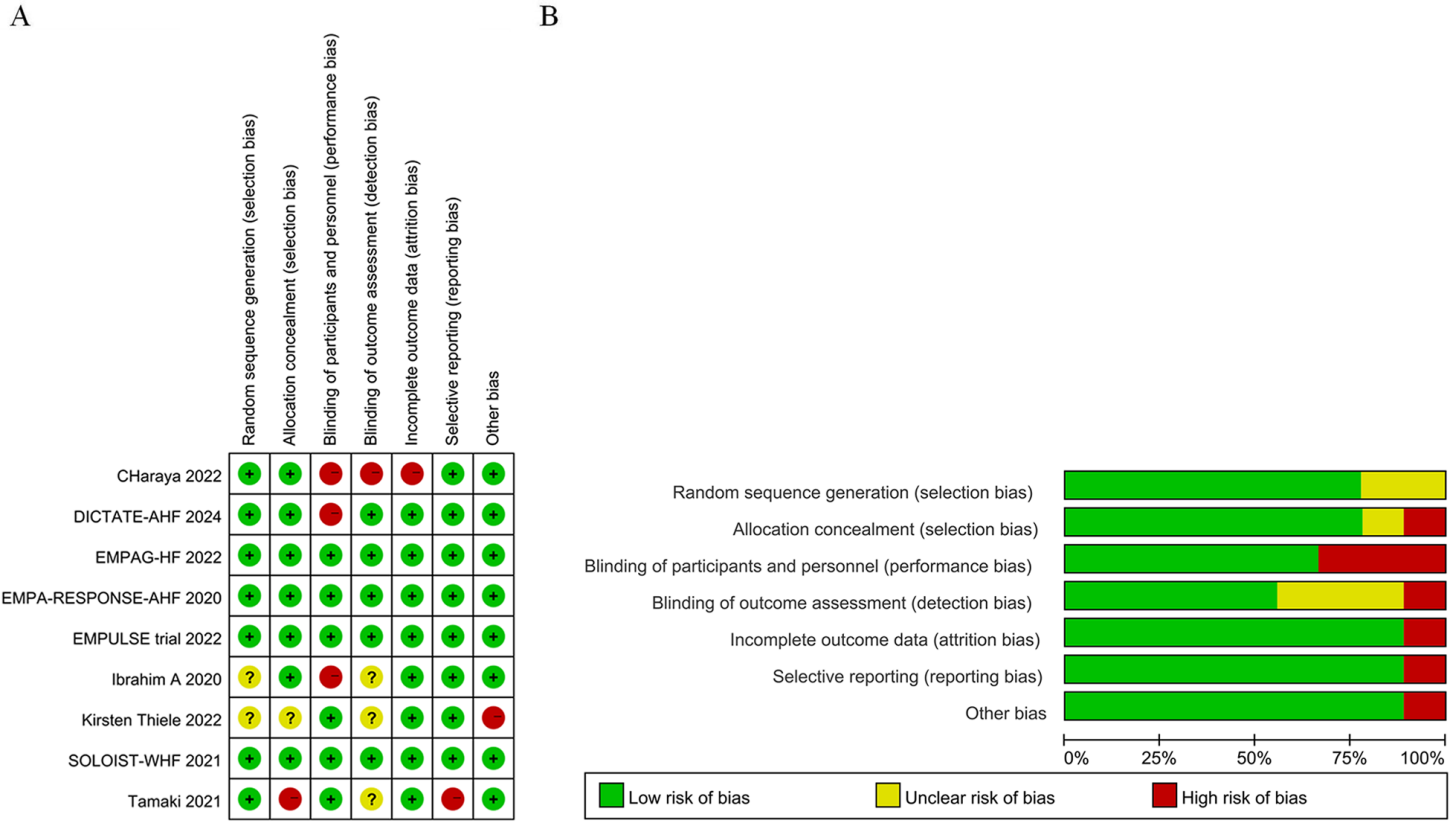
Quality assessment results of included randomized clinically controlled trials (RCT) studies. **(A)** Potential risk of bias for each included study; **(B)** Total risks included in the study.

All included studies had a low risk of bias (Figures 2A,B, Supplementary Table 2).

### 3.3 All-cause mortality

**Figure 3:**
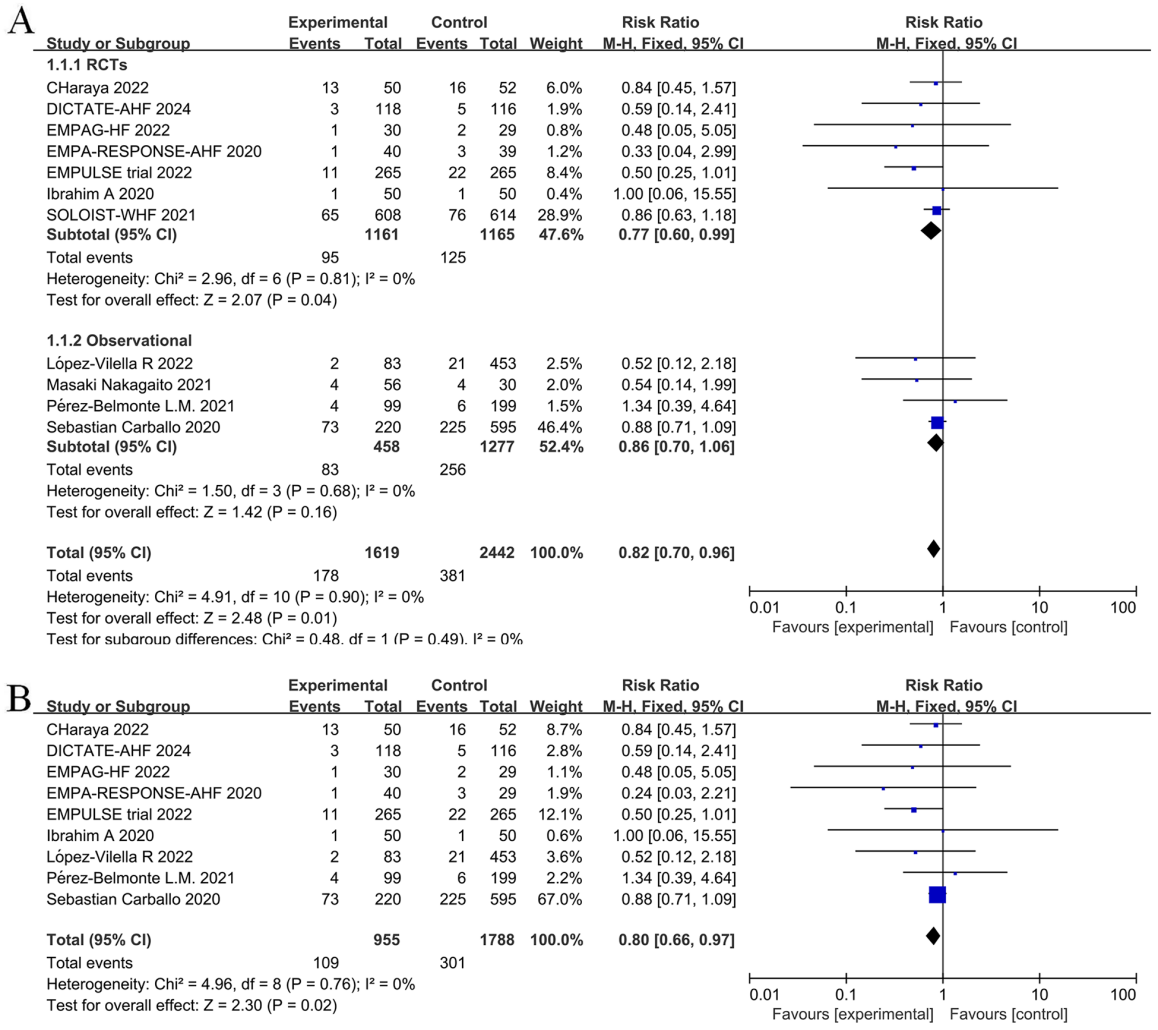
Forest plot for all-cause mortality in patients with acute heart failure (AHF) receiving sodium-glucose cotransporter 2 (SGLT2) inhibitors or placebo. **(A)** Forest plot for all-cause mortality; **(B)** Sensitivity analysis of all-cause mortality in studies where SGLT2 inhibitors treatment was initiated in a hospital.

Given that the longest follow-up period was nine months in the nine randomized controlled studies, we aimed to reduce bias linked to uncertain factors such as follow-up time. Thus, we selected the 12-month results, which represented a relatively short follow-up time, when extracting outcome events from an observational study by Carballo (20). According to our pooled findings of randomized controlled trials and observational studies, long-term treatment with a SGLT2 inhibitor, either during or post-hospitalization, lowers all-cause mortality in patients with AHF (RR = 0.82, 95% CI: 0.70–0.96, *P* = 0.01 < 0.05, I^2^ = 0%, Figure 3A). Compared to the control group, the intervention group showed significant differences in relative risk of death from any cause (RR = 0.77, 95% CI: 0.60–0.99, *P* = 0.04) and absolute risk of death of any cause [risk difference (RD) = −0.02, 95% CI: −0.05–0.00, *P* = 0.04] when only RCTs were included in the meta-analysis. In other words, there was an average of 20 fewer fatalities per 1,000 patients treated with SGLT2 inhibitors in comparison to the control group (with a range of 0 to 50 more fatalities in the control group). While SGLT2 inhibitors were administered to 51.2% of participants after hospital discharge in the SOLOIST-WHF (10) trial. In an observational study by Nakagaito (11), adherence to the SGLT2 inhibitor treatment was categorized into two groups at discharge. However, in the other studies, the intervention group received SGLT2 inhibitors during hospitalization. Therefore, we excluded these two studies and performed a sensitivity analysis across the remainder of the studies, which yielded a statistically significant reduction in relative risk of death in the intervention group compared to the control group (RR = 0.80, 95% CI: 0.66–0.97, *P *= 0.02) (Figure 3B).

### 3.4 Readmission rate

**Figure 4:**
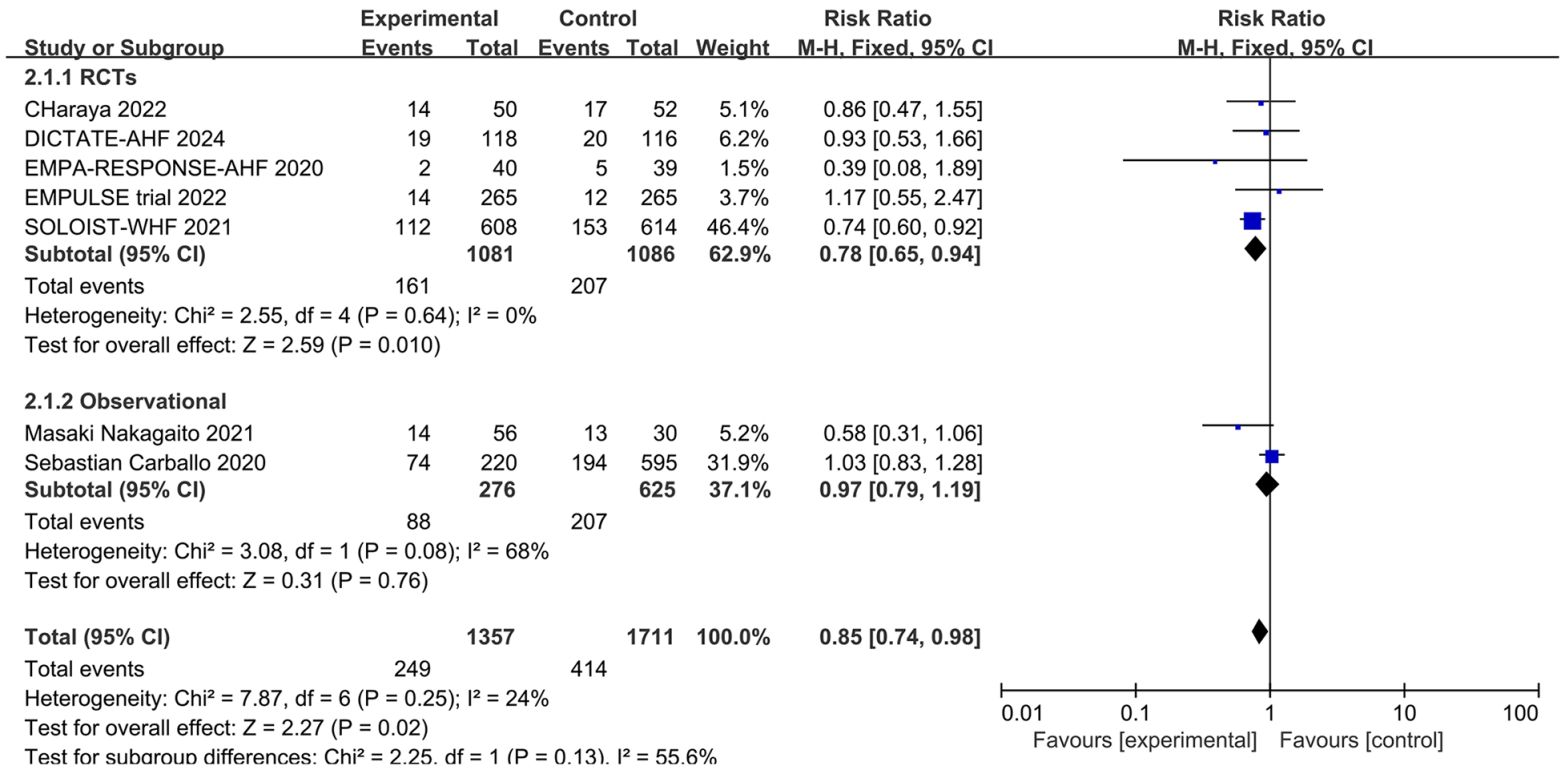
Forest plot of readmission rates in patients with AHF receiving SGLT2 inhibitors or placebo.

Readmission rates were reported in five RCTs and two observational studies. The endpoints for readmission were set at 30 days for DICTATE-AHF (8), EMPULSE (13), and Charaya (14); 60 days for EMPA-RESPONSE-AHF (7); 9 months for SOLOIST-WHF (10); and 12 months for two observational studies (18, 19). Meta-analysis of these seven studies showed that intervention with a SGLT2 inhibitor was associated with a significantly reduced risk of the first readmission post-discharge (RR = 0.85, 95% CI: 0.74–0.98, *P *= 0.02, I^2 ^= 24%, low heterogeneity) (Figure 4). The large heterogeneity among the observational studies did not influence the results.

### 3.5 Cardiovascular events

#### 3.5.1 Number of HFE events

**Figure 5:**
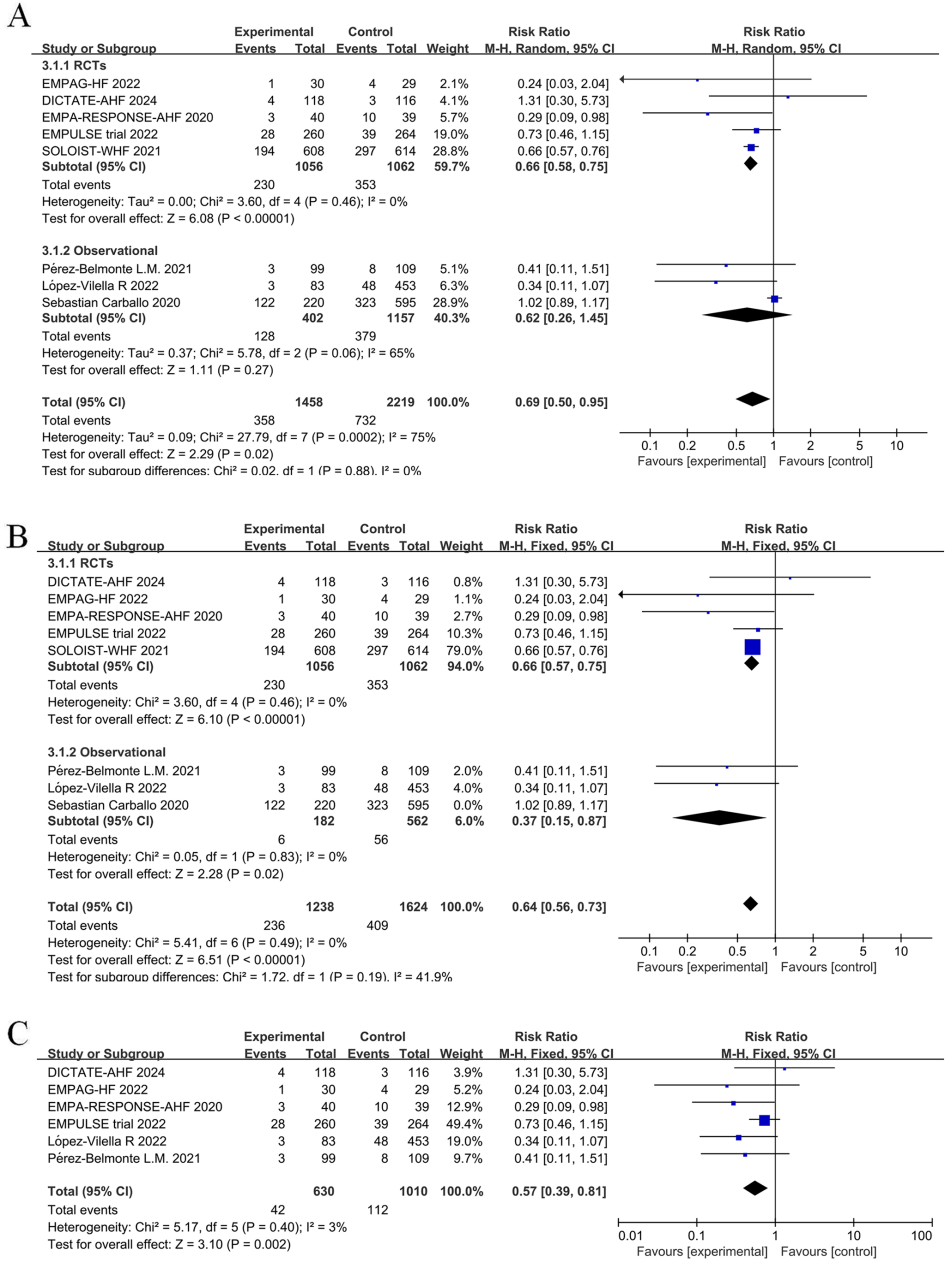
Forest plot and sensitivity analysis of the number of heart failure (HF) exacerbation events. **(A)** Forest plot of the number of HF exacerbation events in patients with AHF; **(B)** Sensitivity analysis; **(C)** Subgroup analysis of studies with in-hospital treatment initiation with or without SGLT2 inhibitors.

Five RCTs and three observational studies reported the number of HFE events among study participants. When combining these studies, the number of HFE events post-discharge in patients with AHF was significantly reduced in the SGLT2 inhibitors group (RR = 0.69; 95% CI: 0.50–0.95, *P* = 0.02 < 0.05, I^2 ^= 75%) (Figure 5A).

#### 3.5.2 Number of rehospitalization events due to HF

**Figure 6:**
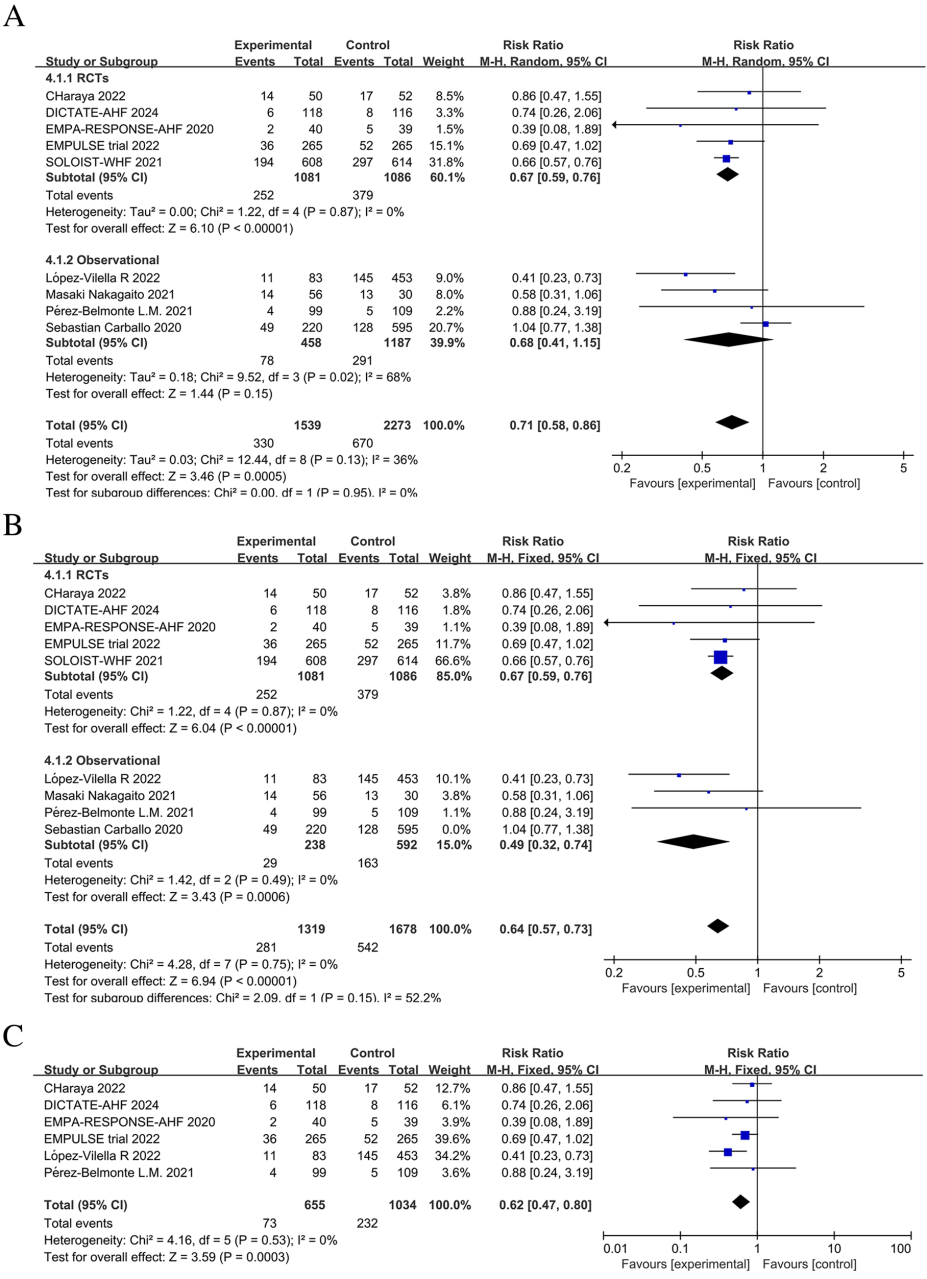
Forest plot and sensitivity analysis of the number of rehospitalization events due to heart failure. **(A)** Forest plot of the number of rehospitalizations due to heart failure in patients with AHF; **(B)** Sensitivity analysis; **(C)** Subgroup analysis of studies with in-hospital treatment initiation with or without SGLT2 inhibitors.

From combining nine studies, it was shown that SGLT2 inhibitors therapy substantially decreased the frequency of rehospitalization among AHF patients as a result of worsening in HF (RR = 0.71, 95% CI: 0.58–0.86, *P* < 0.05, I^2^ = 36%) (Figure 6A). However, there was substantial heterogeneity among studies in the number of HFE events (I^2 ^= 75% >50%). In addition, separately combining the observational studies also showed high heterogeneity. Therefore, we performed a sensitivity analysis of the results, iteratively excluding one study at a time to ensure that the statistically significant reduction in HFE and rehospitalization events in the SGLT2 inhibitors group was not reliant on a single study. The results showed that excluding the Carballo 2020 study (20) eliminated heterogeneity (to 0%) for both HFE events and rehospitalizations due to HF. Sensitivity analysis excluding the Carballo 2020 study (20) showed that use of SGLT2 inhibitors significantly decreased the number of HFE events (RR = 0.64, 95% CI: 0.56–0.73, *P* < 0.05, I^2^ = 0%) and the number of rehospitalization events (RR = 0.64, 95% CI: 0.57–0.73, *P* < 0.05, I^2^ = 0%) (Figures 5B, 6B). To obtain more reliable results, we excluded two studies that grouped patients after discharge, SOLOIST-WHF (10) and Nakagaito (11). This sensitivity analysis showed a 43% reduced risk of HFE events in the SGLT2 inhibitors group compared to the control (RR = 0.57, 95% CI: 0.39–0.81, *P* = 0.002, I^2^ = 3%) (Figure 5C). Exclusion of the SOLOIST-WHF (10) trial, the Carballo (2020) study (20), and the trial by Nakagaito et al. (11) resulted in a statistically significant difference in readmissions due to HF in the SGLT2 inhibitors group compared to the control group (RR = 0.62, 95% CI: 0.47–0.80, *P* < 0.05, I^2^ = 0%) (Figure 6C). The inclusion of observational studies in this meta-analysis did not affect the final results. Overall, this meta-analysis showed that the use of SGLT2 inhibitors, whether initiated at admission or discharge, significantly reduced the occurrence of HFE as well as the number of rehospitalization events due to HF.

#### 3.5.3 Number of deaths due to cardiovascular causes

**Figure 7:**
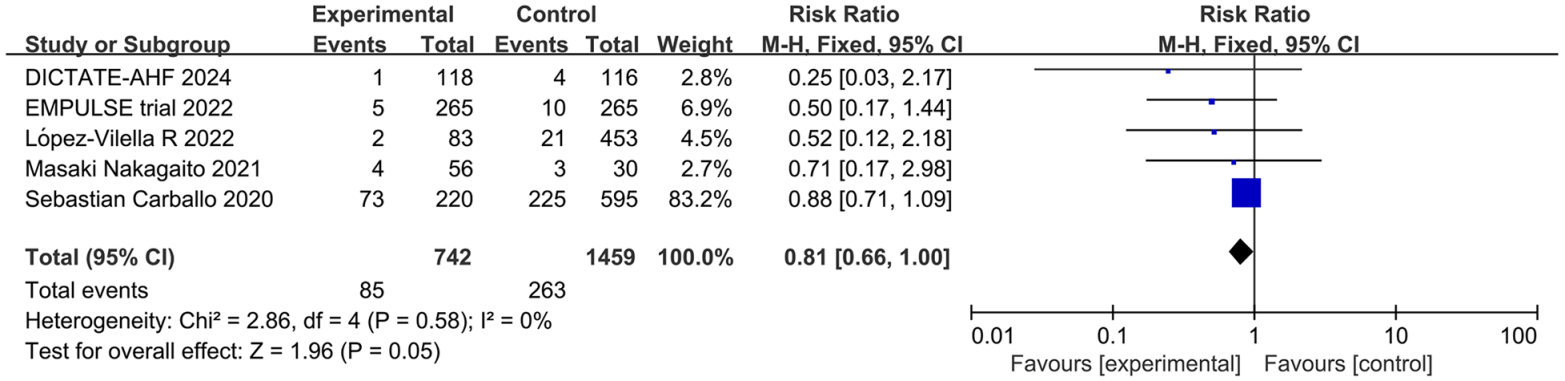
Forest plot of the number of deaths due to cardiovascular causes in patients with AHF.

Meta-analysis of 5 studies showed a statistically significant reduction in the relative risk of death due to cardiovascular disease among patients treated with SGLT2 inhibitors compared to patients not treated with SGLT2 inhibitors (RR = 0.81; 95% CI: 0.66–1.00; *P* = 0.05; I^2 ^= 0%) (Figure 7).

### 3.6 Quality of life assessment

**Figure 8:**
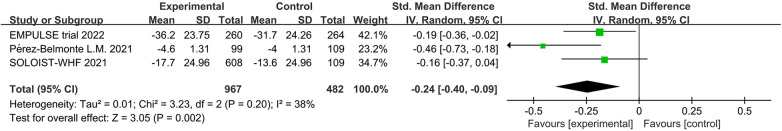
Forest plot of patient quality of life scores.

The EMPULSE trial (13) and the SOLOIST-WHF (10) assessed the impact of SGLT2 inhibitors on patient symptoms, physical activity limitation, and quality of life by administering the Kansas City Cardiomyopathy Questionnaire Total Symptom Score (KCCQ-TSS). Consequently, we evaluated symptoms and health-related quality of life (HRQL) in HF patients using the commonly used KCCQ (21). In addition, the EMPA-RESPONSE-AHF (7), EMPAG-HF (12), and Perez-Belmonte (19) trials assessed patient health status using the visual analogue scale for dyspnea (VAS). The EMPA-RESPONSE-AHF (7) trial showed that treatment with a SGLT2 inhibitor was associated with a lower VAS score for dyspnea than the control group from baseline to day 4; however, the difference was not statistically significant (*P *= 0.18). Similarly, the EMPAG-HF (12) and Perez-Belmonte (19) studies showed larger absolute improvements in health status assessed using the VAS in the SGLT2 inhibitors group compared to the control group; however, these differences were not statistically significant. Using a five-point Likert scale (5PLS) to measure patients’ dyspnea, Ibrahim's study (15) showed a statistically significant improvement in the intervention group compared with the control group (*P* < 0.002). By extracting the pre- and post-treatment differences between the groups and combining the KCCQ and VAS scores for a unified quality of life analysis, we found that patients receiving SGLT2 inhibitors had significantly lower post-operative scores indicating the improved post-operative quality of life among patients in the SGLT2 inhibitors group (SMD = −0.24, 95% CI: −0.40 to −0.09, *P *= 0.002, I^2 ^= 38%) (Figure 8).

### 3.7 Evaluation of kidney-related indicators

**Figure 9:**
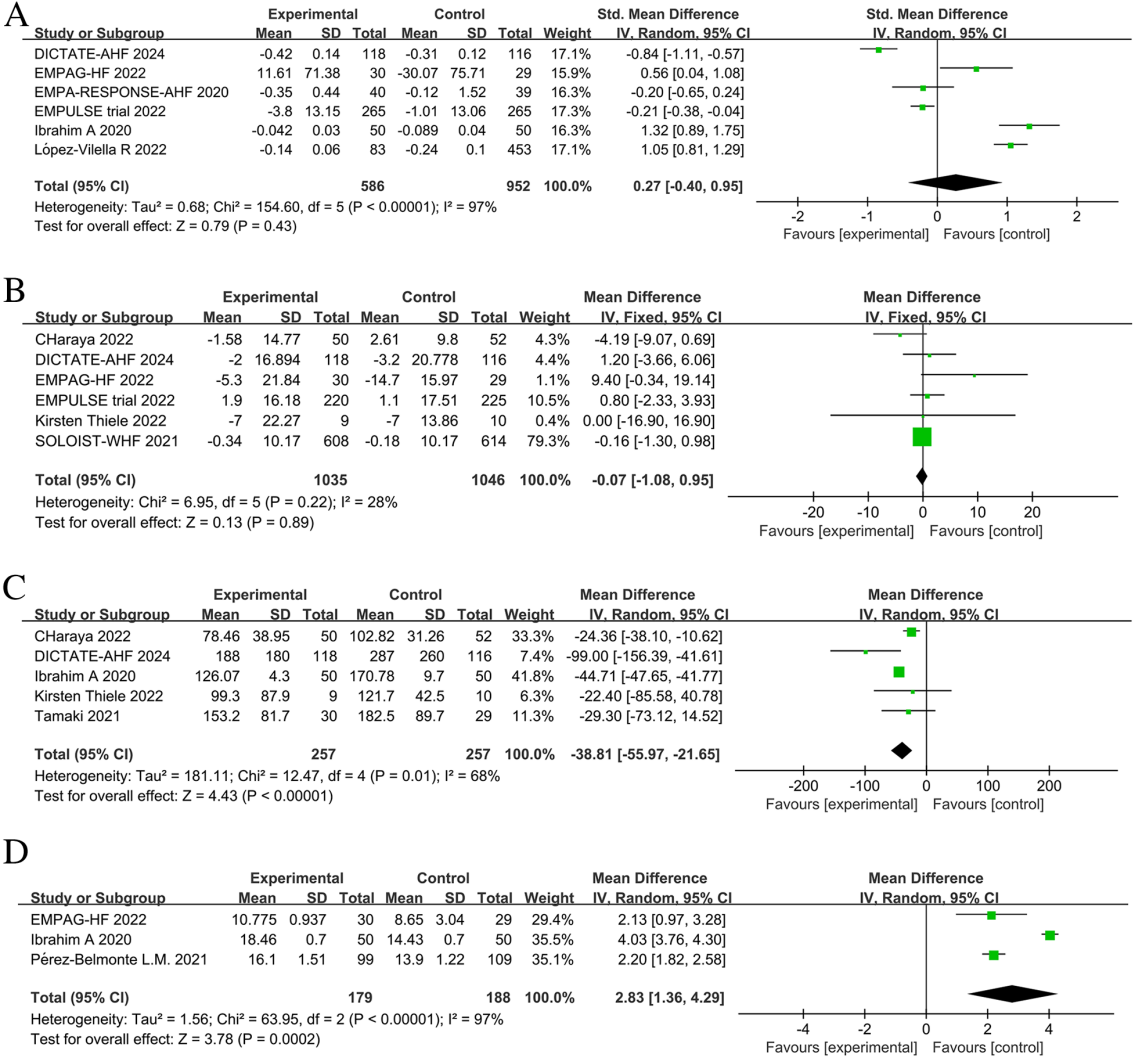
Forest plot of renal-related parameters. **(A)** Diuretic response (kg/40 mg furosemide equivalent); **(B)** estimated glomerular filtration rate (eGFR) (ml/min per 1.73 m^2^); **(C)** 24-hour total furosemide dose (mg); **(D)** Cumulative urine output during hospitalization.

**Figure 10:**
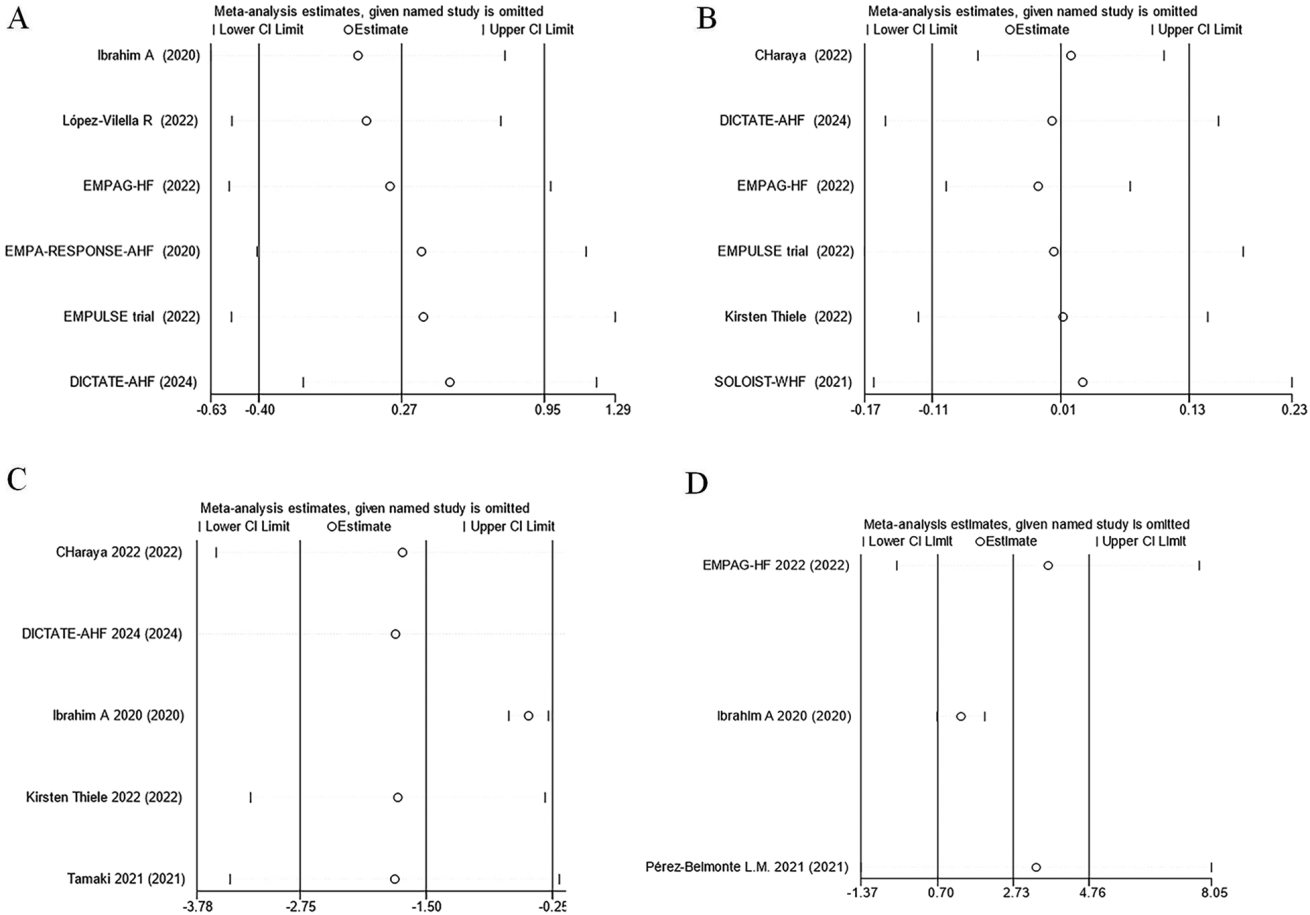
Sensitivity analysis. **(A)** Diuretic response (kg/40 mg furosemide equivalent); **(B)** eGFR (ml/min per 1.73 m^2^); **(C)** 24-hour total furosemide dose (mg); **(D)** Cumulative urine output during hospitalization.

**Figure 11:**
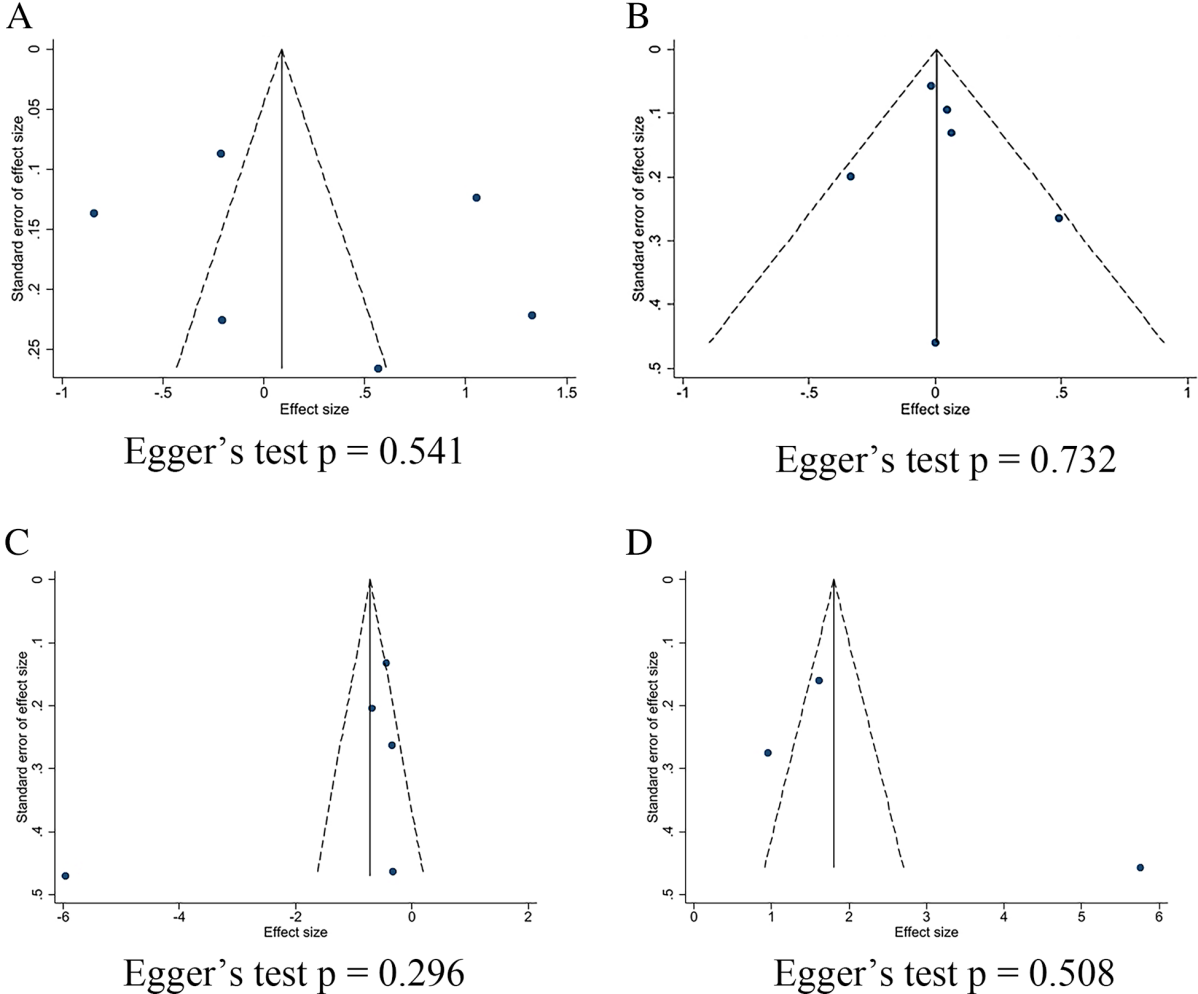
Funnel plots and Egger's test for kidney-related indicators. **(A)** Diuretic response (kg/40 mg furosemide equivalent); **(B)** eGFR (ml/min per 1.73 m^2^); **(C)** 24-hour total furosemide dose (mg); **(D)** Cumulative urine output during hospitalization.

The effect of SGLT2 inhibitors on the diuretic response in patients with AHF remains unclear. The EMPAG-HF (12) trial used ml/mg furosemide equivalents to measure diuretic efficiency, whereas the other trials used kg/40 mg furosemide equivalents. The EMPAG-HF (12), Ibrahim (15), and López-Vilella 2022 (18) trials demonstrated a significant difference in diuretic efficiency between the SGLT2 inhibitors and placebo groups (*P *< 0.05). However, there was no difference detected between the placebo and empagliflozin groups in terms of the diuretic response on day 4 of the EMPA-RESPONSE-AHF (7) trial (*P* = 0.37). Similarly, there was no difference in the OR for improved diuretic efficiency between dapagliflozin and usual care in DICTATE-AHF 2024 (8). In combining the data of diuretic efficiency, a random-effects model suggested that, while the SGLT2 inhibitors group demonstrated higher diuretic efficiency than the placebo group, the difference was not statistically significant (SMD = 0.27, 95% CI: −0.40–0.95, *P* = 0.43, I^2^ = 97%) (Figure 9A). Patients treated with the SGLT2 inhibitor Dagliagazine exhibited a statistically significant reduction in estimated glomerular filtration rate (eGFR) throughout the observation period, in the Charaya 2022 (14) trial (*P* = 0.049). Conversely, the EMPAG-HF (12) trial revealed no variation in eGFR over the course of treatment between the two groups (*P* = 0.598). Furthermore, at six hours, one day, three days, and seven days, Thiele (17) did not observe any statistically significant variation in the eGFR between the treatment and control groups. A comparison of the eGFR research results between the groups showed no statistically significant decline in eGFR (MD = −0.07, 95% CI: −1.08–0.95, *P* = 0.89, I^2 ^= 28%) (Figure 9B). However, treatment with a SGLT2 inhibitor led to a statistically significant decrease in 24-hour total furosemide dose (MD = −38.81, 95% CI:−55.97 to −21.65, *P* < 0.05, I^2^= 68%) and an increase in cumulative urine output during hospitalization in patients with AHF (MD = 2.83, 95% CI: 1.36–4.29, *P* < 0.001, I^2^= 97%) (Figures 9C,D). Sensitivity analysis showed that no single factor significantly affected the results of this meta-analysis, indicating the stability of this study (Figure 10). There was no evidence of bias across trials by visual inspection of funnel plots and using Egger's test for diuretic response (*P* = 0.541 > 0.05), eGFR (*P* = 0.732 > 0.05), 24-hour total furosemide dose (*P* = 0.296 > 0.05), and accumulated urine volume during hospitalization (*P* = 0.508 > 0.05) (Figure 11).

### 3.8 N-terminal pro-brain natriuretic peptide levels

**Figure 12:**
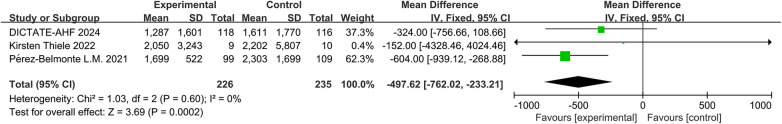
Forest plot of serum N-terminal Pro-brain natriuretic peptide (NT-proBNP) change in patients.

The EMPA-RESPONSE-AHF 2020 (7) trial indicated that although the reduction in NT-proBNP at day 4 was greater with empagliflozin than with placebo, the difference was not statistically significant (*P* = 0.63). Similarly, in the DICTATE-AHF trial (8), although the decrease in NT-proBNP levels was 4% larger in the dapagliflozin group (median: 47% reduction) than in the usual care group (median: 43% reduction), this difference was not statistically significantly different. The EMPULSE trial 2022 (13) and Thiele (17) demonstrated a greater reduction in NT-proBNP concentrations on day 30 in patients receiving empagliflozin than in those receiving placebo; however, it was not stated whether this difference was statistically significant in either trial. In the EMPAG-HF (12), Tamaki (16), Pérez-Belmonte 2021 (19), and Nakagaito (11) trials, compared to the placebo, there were significantly larger decreases in mean NT-proBNP from baseline in the empagliflozin group compared to the control group. Combining the patients’ NT-proBNP levels at the final post-treatment follow-up revealed that the SGLT2 inhibitors-treated group had significantly lower NT-proBNP levels than the control group (MD = −497.62, 95% CI: −762.02 to −233.21, *P* < 0.001, I^2^ = 0%) (Figure 12).

### 3.9 Safety outcomes

**Figure 13:**
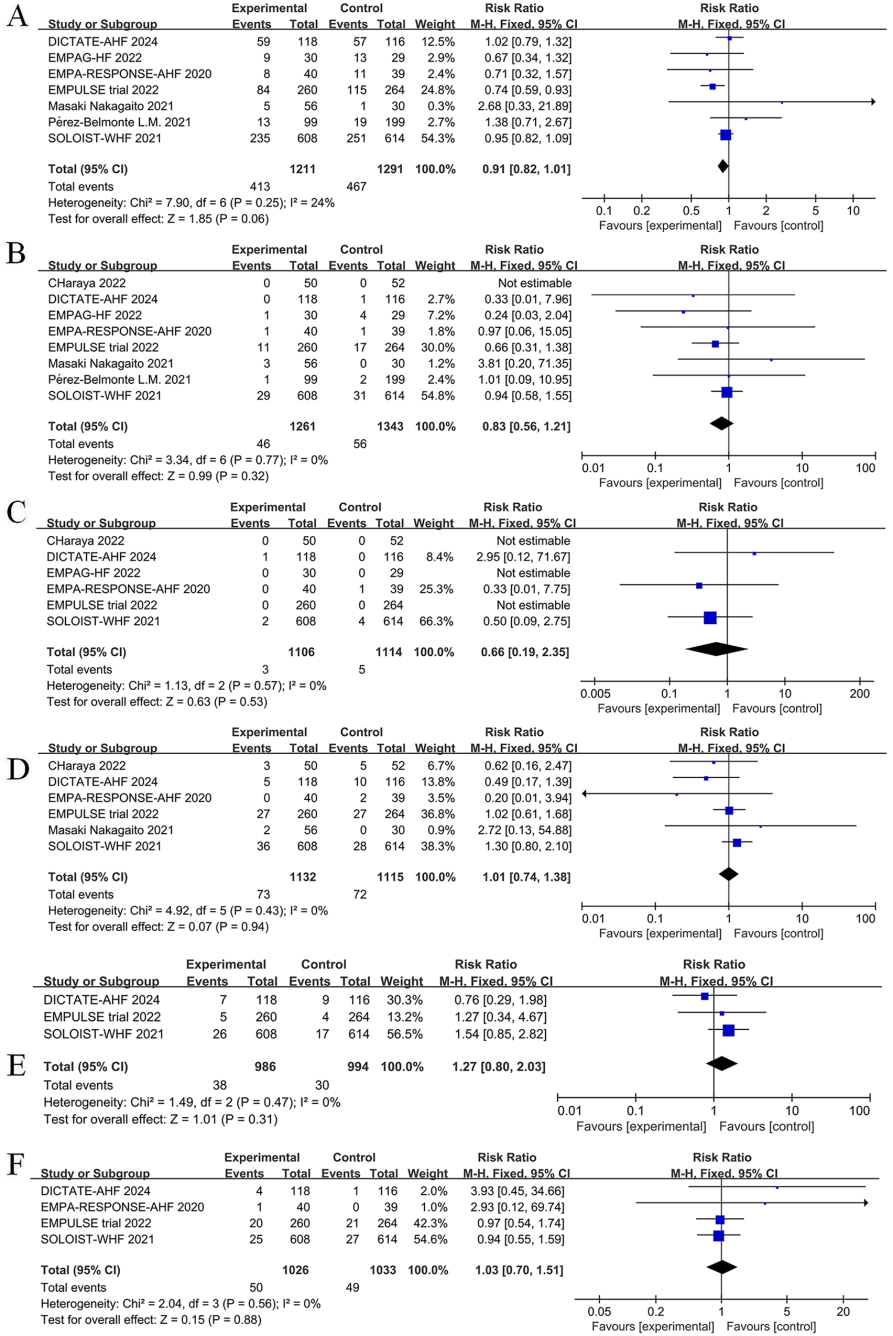
Forest plot of adverse events for SGLT2 inhibitors compared to controls. **(A)** Total number of adverse events; **(B)** Urinary tract infection; **(C)** diabetic ketoacidosis (DKA); **(D)** Hypotension; **(E)** Hypoglycemia; **(F)** Acute kidney injury (AKI).

The impact of SGLT2 inhibitors on adverse events among patients with AHF is displayed in Figure 13. Intervention with SGLT2 inhibitors was not associated with a statistically significant difference in the incidence of adverse events (RR = 0.91, 95% CI: 0.82–1.01, *P* = 0.06, I^2 ^= 24%) (Figure 13A), risk of urinary tract infections (RR = 0.83, 95% CI: 0.56–1.21, *P* = 0.32, I^2 ^= 0%) (Figure 13B), risk of diabetic ketoacidosis (RR = 0.66, 95% CI: 0.19–2.35, *P *= 0.53, I^2 ^= 0%) (Figure 13C), risk of hypotension (RR = 1.01, 95% CI: 0.74–1.38, *P* = 0.94, I^2^ = 0%) (Figure 13D), risk of hypoglycemia (RR = 1.27, 95% CI: 0.80–2.03, *P* = 0.31, I^2^ = 0%) (Figure 13E), or risk of acute kidney injury (AkI) (RR = 1.03, 95% CI: 0.70–1.51, *P *= 0.88, I^2 ^= 0%) (Figure 13F). Overall, current data support the safe use of SGLT2 inhibitors for the management of AHF.

## 4 Discussion

Prevention and treatment strategies for HF have evolved significantly over the past two decades. SGLT2 inhibitors are increasingly recognized for their role in reducing the risk of HF and improving patient prognosis. Several RCTs (21, 22) and a meta-analysis (23) have demonstrated that the use of SGLT2 inhibitors lowers cardiovascular mortality and reduces hospitalizations due to HF in patients irrespective of the presence of diabetes. In addition, researchers have evaluated the quality of life and functional ability of patients with HF. Gao et al. (24) conducted a meta-analysis of RCTs related to SGLT2 inhibitors and HF and which involved formal assessments of patients’ functional capacity. They showed that the use of SGLT2 inhibitors is associated with improvement in patient outcomes, as measured by objective assessments of maximal exercise capacity and validated quality of life questionnaires, regardless of sex or ejection fraction. The most recent and largest meta-analysis on HF patients and SGLT2 inhibitors was conducted by Mohammed Tarek Hasan et al. (25). This meta-analysis categorized patients with HF based on the presence or absence of diabetes and showed that SGLT2 inhibitors significantly lowered the risk of hospitalizations for heart failure (HHF) among patients with and without diabetes; however, there was no statistically significant difference in cardiovascular mortality or serious adverse events.

The most recent HF guidelines in the United States and Europe recommend that first-line treatment for patients with HF with reduced ejection fraction (HFrEF) or HF with preserved ejection fraction (HFpEF) should include SGLT2 inhibitors and diuretics (26). Large clinical studies, such as the EMPEROR-Preserved (27) and DELIVER trials (28), have shown that empagliflozin and dapagliflozin reduce cardiovascular death and HHF in patients with HFrEF or HFpEF, irrespective of diabetes status, when added to a standard HF regimen. Furthermore, the findings of a meta-analysis suggest that SGLT2 inhibitors can significantly reduce all-cause mortality, cardiovascular death, and the incidence of first hospitalization for HF in patients with HFrEF and HFpEF (29). However, the effect of SGLT2 inhibitors on patients with AHF remains contentious. Thus, our meta-analysis aimed to evaluate the efficacy of SGLT2 inhibitors specifically in patients with AHF.

We reviewed several relevant studies on the effectiveness of SGLT2 inhibitors in patients with AHF. Notably, the meta-analyses conducted by Salah (30) and Amin (31) in 2022 focused only on three RCTs, whereas Patoulias included six RCTs (32). However, Carvalho's (33) meta-analysis incorporated the DELIVER trial (28), which did not exclusively include patients with AHF. We excluded the DELIVER trial from our analysis to maintain consistency in the patient population and reduce bias. In addition to RCTs, we considered observational studies in our meta-analysis to ensure a comprehensive evaluation of SGLT2 inhibitors in patients with AHF. By including all available trials, regardless of their study design, we aimed to capture a broader perspective on the topic. The DICTATE-AHF trial (8) showed that early use of dapagliflozin in hospitalized patients with ADHF does not worsen any pre-specified safety outcomes, suggesting that dapagliflozin can be safely initiated upon admission and can be used to rapidly optimize guideline-guided drug regimens. The DICTATE-AHF trial provided additional evidence supporting the prognostic benefits of SGLT2 inhibitors in patients with AHF. Thus, we included the results of this trial in our meta-analysis, resulting in a total sample size of 4,053 patients.

Consistent with previous studies, our findings suggest that long-term treatment with SGLT2 inhibitors, either during hospitalization or after discharge, can lead to a considerable decrease in all-cause mortality. This meta-analysis showed that the mortality risk in the intervention group was 18% lower than in the control group, highlighting the potential life-saving benefits of SGLT2 inhibitors in patients with AHF. To further assess the effect of SGLT2 inhibitors on mortality, we conducted sensitivity analyses according to the study design. Suggested mechanisms by which SGLT2 inhibitors lower the risk of mortality in patients with HF include their anti-inflammatory properties, support of antioxidant defense systems, and reduced cardiac remodeling or fibrosis (34–36). Proposed alternative mechanisms include cardioprotective effects by promotion of ketolysis and ketone body levels (37). Thus, SGLT2 inhibitors may play comparable roles in patients with AHF. Further research is required to investigate how SGLT2 inhibitors lower the mortality rates in individuals with AHF.

Although SGLT2 inhibitors reduce the number of cardiovascular deaths and hospitalizations for HF in patients with HFpEF with or without diabetes, it remains unclear (38, 39) as to whether SGLT2 inhibitors added to the standard HF regime reduce the readmission rate. Our meta-analysis provides insights to this question. The overall analysis showed a statistically significant 15% reduction in the risk of readmissions in the SGLT2 inhibitors group compared to the control group. Furthermore, the reduction in readmission rates observed when all studies were combined suggests that SGLT2 inhibitors have the potential to improve post-discharge outcomes in patients with AHF.

A meta-analysis showed that patients with HF and a decreased ejection fraction who were treated with dapagliflozin had a lower risk of worsening HF and better symptom scores than those who received a placebo, regardless of diabetes status (40). Our analysis also showed a significant reduction in the number of HFE events post-discharge in the SGLT2 inhibitors group compared with the placebo group. This finding is consistent with previous studies that have shown the beneficial effects of SGLT2 inhibitors in reducing the risk of HF events (40–42). Additionally, our meta-analysis revealed a significant decrease in the frequency of rehospitalization events due to HF in patients receiving SGLT2 inhibitors therapy. Sensitivity analysis confirmed the robustness of these results, reinforcing the significant reduction in both HFE events and rehospitalization due to HF resulting from treatment with SGLT2 inhibitors. However, it is important to acknowledge the substantial heterogeneity observed in the combined results of both HFE events and rehospitalization rates. This heterogeneity may be attributed to variations in study design, patient characteristics, or other factors. Sensitivity analyses according to the study type still showed statistically significant reductions in HFE events and rehospitalizations, indicating the consistency of the findings. We additionally evaluated the impact of SGLT2 inhibitors on patient quality of life and showed that patients in the SGLT2 inhibitors group exhibited a significantly improved quality of life compared to those in the control group. Overall, these findings demonstrate the positive effects of SGLT2 inhibitors on symptoms, physical activity limitations, and health-related quality of life in patients with HF.

It has been proposed that SGLT2 inhibitors regulate the interstitial vs. intravascular volume differently from other diuretics. Compared with conventional diuretics, SGLT2 inhibitors facilitate a higher volume of fluid clearance from the interstitial fluid space as opposed to circulation, perhaps leading to an increase in urine production in the SGLT2 inhibitors group (28). Our meta-analysis showed no statistically significant difference in the diuretic response between the SGLT2 inhibitors and control groups. However, SGLT2 inhibitors caused a significant increase in urine volume in patients with AHF, suggesting that SGLT2 inhibitors positively affect diuresis in patients with HF.

In terms of safety outcomes, the analysis indicated that SGLT2 inhibitors were associated with a lower risk of major adverse events than the control group. Additionally, there was no significant difference in the risk of urinary tract infections, hypotension, or hypoglycemia in the SGLT2 inhibitors group. Importantly, the use of SGLT2 inhibitors did not increase the risk of acute kidney injury in patients with AHF.

## 5 Strengths and limitations

Although this study has certain limitations, such as its regional focus and potential selection bias due to small sample sizes in some RCTs, it provides valuable insights into the benefits of SGLT2 inhibitors in patients with AHF. Combining both randomized controlled trials and observational studies may introduce some bias; however, the overall data support the conclusion that treatment with SGLT2 inhibitors in AHF can contribute to reduced mortality and a decrease in the total number of adverse events.

## 6 Conclusion

The findings from this meta-analysis of 13 RCTs and observational studies support the benefit of SGLT2 inhibitors in improving AHF outcomes, including reduced mortality, HF events, and rehospitalizations in patients with AHF. These inhibitors also have positive effects on quality of life and diuresis. Although some safety concerns exist, the overall risk-benefit profile favors using SGLT2 inhibitors in this patient population. Further studies with diverse populations and longer follow-up periods are required to validate and expand upon these findings.

## Data Availability

The original contributions presented in the study are included in the article/Supplementary Material, further inquiries can be directed to the corresponding author.
